# Antibiotic use in Brazilian hospitals in the 21^st^ century: a systematic review

**DOI:** 10.1590/0037-8682-0861-2020

**Published:** 2021-06-09

**Authors:** Lucas Borges Pereira, Maria Olívia Barboza Zanetti, Larissa Pombeiro Sponchiado, João Paulo Vilela Rodrigues, Marília Silveira de Almeida Campos, Fabiana Rossi Varallo, Leonardo Régis Leira Pereira

**Affiliations:** 1 Universidade de São Paulo, Faculdade de Ciências Farmacêuticas de Ribeirão Preto, Departamento de Ciências Farmacêuticas, Ribeirão Preto, SP, Brasil.

**Keywords:** Drug utilization, Pharmacoepidemiology, Anti-bacterial agentes, Drug resistance, Bacteria, Antimicrobial stewardship

## Abstract

**INTRODUCTION:**

This systematic review aimed to assess antibiotic use in Brazilian hospitals in the 21st century, as well as to understand the different drug utilization metrics adopted to assess the consumption of these drugs.

**METHODS:**

We systematically reviewed five databases (MEDLINE [Medical Literature Analysis and Retrieval System Online], CENTRAL [The Cochrane Central Register of Controlled Trials], EMBASE® [Excerpta Medica Database], Scopus [Elsevier’s abstract and citation database], and LILACS [*Literatura Latino-Americana e do Caribe em Ciências da Saúde*]) for observational or experimental studies that assessed antibiotic utilization in Brazilian hospitals. The main outcomes were the drug utilization metrics and the consumption of antibiotics.

**RESULTS:**

We included 23 studies, of which 43.5% were carried out in adult and pediatric care units, 39.1% in adult units, and 17.4% in pediatric units. Regarding the complexity of healthcare, 26.1% of the studies were performed in intensive care units. Two drug utilization metrics were used in these studies: the defined daily dose (DDD) and the percentage of antibiotic prescriptions. The most commonly used antibiotic classes were third-generation cephalosporins, carbapenems, fluoroquinolones, and combinations of penicillins when the DDD was the adopted drug utilization metric.

**CONCLUSIONS:**

Although few studies have been conducted, existing data indicate a high use of broad-spectrum antibiotics. We found that the lack of standardized antibiotic utilization metrics impaired the mapping of drug consumption at the national level.

## INTRODUCTION

Since the discovery of penicillin in 1928, antibiotics have become an important factor associated with an epidemiological transition characterized by a drastic reduction in morbidity and mortality from infectious diseases and a concomitant increase in the prevalence of chronic diseases^1^. However, antibiotic use promotes the selection of resistant bacteria, which is a recognized public health problem. Appropriate use of these drugs reduces bacterial resistance to traditional therapies; therefore, it is essential in mitigating this problem^2^ ^-^ ^4^.

Because multidrug-resistant bacteria have become increasingly prevalent, the available therapeutic arsenal is ineffective in treating some infectious diseases^5^. Moreover, recent studies show that changes in human microbiota, which may be a consequence of antibiotic use, are an important factor in the development of several diseases such as obesity, diabetes, and allergic diseases, among others^6^.

To avoid possible harmful consequences arising from inappropriate antibiotic use, developed countries and health organizations have developed recommendations and surveillance policies to promote the rational use of antibiotics, including the Strategies to Address Antimicrobial Resistance Act^7^, European Surveillance of Antimicrobial Consumption Network^8^, and Global Antimicrobial Resistance Surveillance System linked to the World Health Organization (WHO)^9^. These initiatives aim to monitor antibiotic use, identify drug-related problems, and propose strategies to promote rational drug use.

In Brazil, antibiotic consumption data are generally obtained from local and regional studies focusing mainly on hospital use^10^. Most of these studies focused on the use of only some antibiotics^11^ or a specific hospital unit^12^.

Considering the absence of a national Brazilian study, studies on antibiotic use in local and regional hospitals have become essential for the description and analysis of antibiotic consumption in Brazil and to propose strategies to optimize their use. We aimed to synthesize the existing studies by conducting a systematic review to assess antibiotic use and consumption in Brazilian hospitals in the 21st century and to understand the different drug utilization metrics adopted to assess the consumption of these drugs.

## METHODS

A systematic literature review was conducted in accordance with the Preferred Reporting Items for Systematic Reviews and Meta-Analyses guideline^13^ and criteria established by the Cochrane Collaboration^14^ previously registered with the International Prospective Register of Systematic Reviews (identification code CRD42020153154).

Publications that answered the guiding question: “What is the pattern of antibiotic consumption in Brazilian hospitals?” were selected. Observational and experimental studies performed in Brazilian hospitals that analyzed the general pattern of antibiotic consumption, consumption of specific classes, or use according to a specific purpose (empirical or targeted treatment or prophylaxis) in the entire hospital or in specific care units were included. Articles were excluded for the following factors:

- data was collected before the beginning of the 21st century;
- conceptually described/discussed consumption of antibiotics in Brazil, comments or expert opinions, study protocols, reviews (narrative, integrative, and systematic), dissertations or theses, editorials, news reports, and abstracts published in conference proceedings;
- published in languages other than English, Portuguese, or Spanish;
- did not have full text available.

The search strategy construction incorporated terms that characterized the guiding question, structured by the PICOS acronym (Population: hospitalized patients in Brazil; Intervention: use of antibiotics; Comparison: not applicable; Outcomes: consumption of antibiotics and drug utilization metrics applied; Study type: observational or experimental)^15^. 

This strategy was developed using a combination of terms from the Medical Subject Headings (MeSH) thesaurus of the PubMed database (MEDLINE [Medical Literature Analysis and Retrieval System Online]). The MeSH terms selected are descriptors of each PICO component: anti-infective agents, antibacterial agents, hospitals, and Brazil. For each MeSH term, relevant keywords were selected to expand the search and were combined with the Boolean operator “OR” as follows:

1. anti-infective agents OR antibacterial agents OR antimicrobial* OR antibiotic* OR antimicrobial OR antibacterial agents [Pharmacological Action];
2. hospital OR hospital*;
3. Brazil.

The terms used in 1, 2, and 3 described above were combined using the Boolean operator “AND.” 

This database has been adapted for other databases, namely CENTRAL (The Cochrane Central Register of Controlled Trials), EMBASE® (Excerpta Medica Database), Scopus (Elsevier’s abstract and citation database), and LILACS (*Literatura Latino-Americana e do Caribe em Ciências da Saúde*). The search was conducted in April 2019.

Article selection was carried out using the Rayyan systematic review application - a web and mobile app for systematic reviews^16^. Duplicates were removed. The reading and critical analysis of the publication titles and summaries began according to the inclusion and exclusion criteria to screen articles of interest for further evaluation. To avoid screening bias, two researchers (LBP and MOBZ) were designated to carry out the aforementioned steps independently. Disagreements between researchers were resolved through discussion and consensus. 

After screening potential publications, the remaining articles were analyzed in full by the same researchers. At this stage, articles were defined according to the inclusion criteria and were incorporated into the systematic review. A manual search was performed using the reference lists of the selected articles to identify other relevant publications that were not identified in the databases. 

Finally, two articles were randomly chosen for a pilot test of the data extraction form that was built based on the recommendations from the Cochrane Reviewers’ Handbook^14^. Subsequently, the data extracted on the variables of interest for each article were organized in a table.

The quality of the included studies was assessed using the 2018 Mixed Methods Appraisal Tool (MMAT), a critical appraisal designed to assess systematic reviews of mixed studies. It appraises the methodological quality of five categories: qualitative research, randomized controlled trials, nonrandomized studies, quantitative descriptive studies, and mixed methods studies. There is no overall score because it does not provide information on what aspects of the studies are problematic. When the study receives a “yes” to any item of this tool, it means that the category assessed adequately meets the criteria. Conversely, “no” means that the category does not adequately meet the criteria and “can’t tell” means that the category does not report sufficient information to answer “yes” or “no” according to the criteria^17^ ^,^ ^18^.

The study hospitals were classified according to their sizes^19^: small (up to 50 beds), medium (51 to 150 beds), large (151 to 500 beds), and extra-large (more than 500 beds). If a local study was conducted in an intensive care unit (ICU), it was classified as an ICU, and if it was conducted in a pediatric ward and/or pediatric ICU, it was classified as pediatrics. 

The included articles presented two antibiotic drug utilization metrics: the relative frequency of antibiotic prescriptions (percentage of specific antibiotic prescribed divided by the total number of antibiotic prescriptions or percentage of patients treated with a specific antibiotic divided by the total number of patients treated with antibiotics) and the defined daily dose (DDD/beds-days, DDD/patients-days, or DDD/admissions). The DDD/beds-days, patients-day, or admissions unit was used in some studies divided by 100 and in others by 1,000. Thus, to facilitate the comparison, the studies that divided by 1,000 were divided by 10 to present them as DDD/100 bed-days, patient-days, or admissions. Furthermore, this study, included antibiotics in the consumption analysis classified as J01 in the 2019 Anatomical Therapeutic Chemical (ATC)/DDD index.

If one study presented results of antibiotic consumption from more than one place or for more than one type of treatment (i.e., more than one hospital or ICU or for empiric and adjusted treatment), all results were included in this study as multiple presentations of antibiotic use.

## RESULTS

**FIGURE 1:**
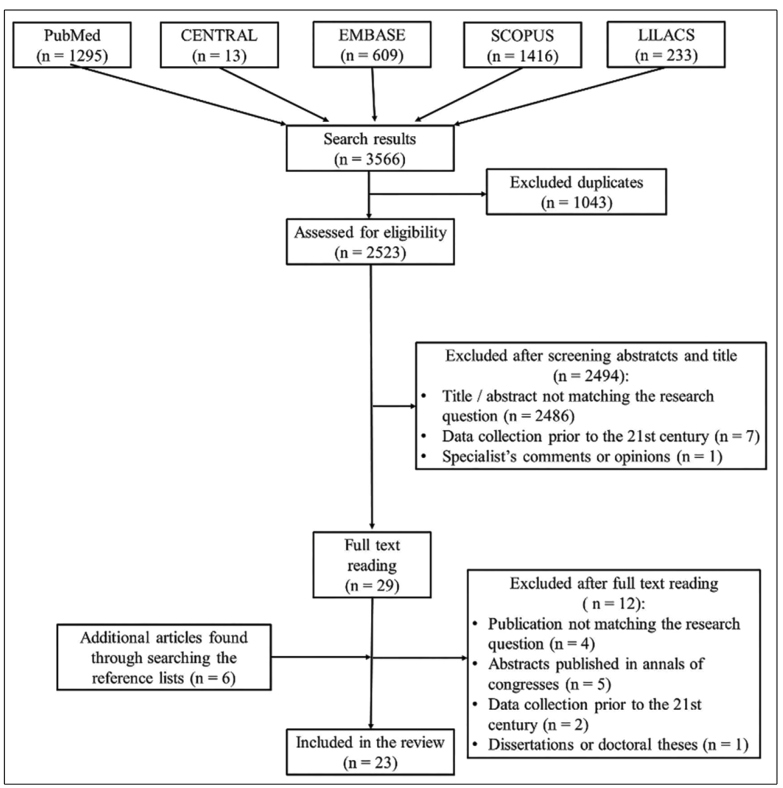
Flowchart of article selection process for systematic review.

After applying the search strategy in the databases, 3,566 publications were selected for title and abstract evaluation. Publications for which there was disagreement were excluded or included after discussion and consensus. Finally, 29 studies were selected for full reading, and 17 were considered suitable for inclusion in the systematic review. By manually searching the reference lists, 6 more articles were included, totaling 23 articles. Figure 1 shows the study selection process.

**CENTRAL:** The Cochrane Central Register of Controlled Trials; **EMBASE®:** Excerpta Medica Database; **LILACS**: *Literatura Latino-Americana e do Caribe em Ciências da Saúde*; **PubMed:** interface used to search MEDLINE (Medical Literature Analysis and Retrieval System Online); **Scopus:** Elsevier’s abstract and citation database.

The 23 included articles were published between 2003 and 2018, of which 69.5% were observational studies (Supplementary Material (Table 1 **)** ^20^ ^-^ ^42^. The data collection periods lasted between 3.5 and 120 months, with an average of 27 months (standard deviation 28.6) and a median of 20 months. Regarding the data collection sites, 43.5% of studies were carried out in adult and pediatric care units, 39.1% in units that care for only adults, and 17.4% that only care for pediatrics. Notably, 26.1% of studies were conducted only in ICUs.

One study presented results of the two previously reported drug utilization metrics (DDD and frequency). Furthermore, 14 (60.9%) studies described antibiotic use in the treatment and prophylaxis of infections, while 9 (39.1%) described only use for treating infections (Supplementary Material (Table 1).

Supplementary Material (Table 2) shows the study characteristics according to the antibiotic assessed, inclusion criteria of patients, and drug utilization metrics applied. Of the 13 studies that used the DDD, only 6 described the calculations and ATC/DDD index version (one, 1999 version; three, 2005; one, 2006; and one, 2010). Of the 13 studies not conducted in ICUs or pediatric units, only 5 described the units where the research took place.

The utilization metrics found in this review were DDD/bed-days in 4 studies, DDD/patient-days in 8, and DDD/100 admissions in 1. Among these, two presented antibiotic consumption data arising from multiple locations (three for dos Santos et al., 2010^22^) and more than one treatment (two for Gimenes et al. 2016^29^), totaling 16 columns as shown in Table 3.

**TABLE 3:** Consumption of antibiotics, classified according to the ATC classification, calculated in Defined Daily Dose (DDD)/100 beds-days or DDD/100 patients-day.

	Small-sized hospitals	Medium-sized hospitals		Large-sized hospitals				Extra-sized hospitals		ICU							
	Entire hospital^28^	Adult ward^20^	Entire hospital^39^	Adult ward^36^	Entire hospital^38^	Entire hospital^41^	Entire hospital^42^	Entire hospital^27^	ICU adult^21^	ICU_1^22^	ICU_2^22^	ICU_3^22^	MSSA^29^	MRSA^29^	ICU^3^	ICU^5^
Tetracycline - J01AA	-	-	-	-	-	-	-	0.4	-	-	-	-	0,1	-	-	-
Amphenicols - J01BA	1.6	-	-	-	-	-	-	-	-	-	-	-	-	-	-	-
Broad spectrum penicillins - J01CA	12.8*	-	2.0	-	-	-	-	9.0#	0.6	-	-	-	-	-	9.5	-
Penicillins and combinations - J01CR	7.5	-	18.9	-	7.6	-	0.7	6.0	53.5	35.1	30.4	69.5	55,2	167,4	8.3	-
Beta lactamase sensitive penicillins - J01CE	12.8*	-	3.6	-	-	-	-	-	3.2	-	-	-	-	-	-	-
Beta lactamase resistant penicillins - J01CF	12.8*	1.4	11.6	-	-	-	-	9.0#	8.4	-	-	-	53,6	17,2	-	-
1^st^ generation cephalosporins - J01DB	34.2	-	11.4	-	-	-	-	8.0	8.1	0.9	1	11.3	-	-	5.7	-
2^nd^ generation cephalosporins - J01DC	-	-	0.0	-	0.1	-	-	1.2	12.1	-	-	-	-	-	3.8	-
3^rd^ generation cephalosporins - J01DD	98.0	10.0	9.6	0.7	6.7	-	6.4	13.5	23.9	43.5	26.4	22.0	30,6	56,4	19.6	55.1
4^th^ generation cephalosporins - J01DE	7.8	-	1.3	-	1.3	-	1.6	8.8	-	-	-	-	39,7	153,1	23.4	
Monobactamics - J01DF	-	-	0.5	-	-	-	-	-	0.7	-	-	-	-	0,2	-	-
Carbapenem - J01DH	2.8	1.3	2.9	1.6	1.1	1.6	1.8	18.4	15.5	25.0	25.7	17.5	31,6	152,1	18.4	26.4
Intermediate-acting sulfonamides - J01EC	-	-	-	-	-	-	-	-	-	-	-	-	-	-	-	-
Combination of sulfonamides and trimethoprim - J01EE	0.7	-	0.1	-	-	-	-	3.9	2.4	7.8	0.3	18.4	-	-	-	-
Macrolides - J01FA	2.0	-	1.1	-	1.5	-	-	8.6	2.2	-	-	-	0,1	14,3	-	-
Lincosamides - J01FF	12.7	2.7	5.9	-	-	-	-	6.1	8.9	16.7	109.0	3.3	25,7	41,1	-	-
Aminoglycosides - J01GB	8.5	6.6	8.5	2.4	-	-	-	2.9	9.5	12.4	6.6	19.0	0,1	1	-	-
Fluoroquinolones - J01MA	42.6	1.6	18.8	2.7	7.4	-	5.8	7.0	21.2	6.6	4.4	20.0	46,6	135,4	8.8	-
Other quinolones - J01MB	-	-	-	-	-	-	-	-	-	-	-	-	-	-	13.6	-
Glycopeptide - J01XA	2.2	1.5	4.8	-	1.0	-	2.3	11.8	14.5	5.9	19.8	12.0	13	117,7	-	27.0
Polymyxins - J01XB	-	-	-	-	0.4	-	0,4	5.8	-	-	-	-	-	-	-	-
Imidazolic derivatives - J01XD	8.7	-	5.1	-	-	-	-	-	-	-	-	-	-	-	-	-
Nitrofuran derivatives - J01XE	-	-	-	-	-	-	-	0.1	-	-	-	-	-	-	-	-
Other antibacterials - J01XX	-	-	1.3	-	-	-	-	1.6	4.1	-	-	-	0,8	77,5	-	-
Total antibiotic utilization	-	-	107,4	-	27,1	-	-	-	188,8	153,9	223,6	193,0	-	-	111,1	-

**ICU adult:** intensive care unit that cares only for adults; **ICU:** intensive care unit; **Entire hospital:** consumption within the entire hospital; **MSSA:** consumption of antibiotics to treat infection caused by methicillin-sensitive *Staphylococcus aureus*; **MRSA:** consumption of antibiotics to treat infection caused by methicillin-resistant *Staphylococcus aureus*. *This value is assigned in the article for penicillins, except penicillins and combinations that have been calculated, # total value of amoxicillin, ampicillin and oxacillin use. Total antibiotic utilization: sum of DDDs of each study which included all antibiotics belonging to J01 ATC classification.

Moreover, 11 studies used the relative frequency of antibiotic prescriptions; among these, one presented antibiotic consumption data of more than one treatment type. One study was not included in Table 4 because the results of antibiotic use were presented by frequency of prescriptions by therapy type (antibiotics prescribed as monotherapy and polytherapy)^32^. In this study, the most frequently prescribed antibiotics were amoxicillin and ampicillin (62.17%), followed by ampicillin plus gentamicin (7.96%), and oxacillin plus ceftriaxone (6.86%). Therefore, the total number of columns in Table 4 regarding antibiotic use is 11. 

**TABLE 4:** Percentage of antibiotic prescriptions in each study which used antibiotics classified according to the ATC classification.

	Small-sized hospitals	Medium-sized hospitals	Large-sized hospitals	Extra-sized hospitals	UTI			Pediatrics			No information
	Entire hospital^23^	Adult ward^26^	Entire hospital^24^	Cl & Sur ward. ICU^40^	ICU^22^	ICU tto emp^37^	ICU tto dir^37^	ICU and Ped ward^25^	ICU and Ped ward^30^	Ped^31^	CM^34^
Tetracycline - J01AA	-	-	-	-	-	-	-	-	1.1	-	-
Amphenicols - J01BA	-	-	-	-	-	-	-	-	1.1	-	-
Broad spectrum penicillins - J01CA	51.0	-	4.0	7.2	-	1.6	3.2	5.0	4.3	90.6	-
Penicillins and combinations - J01CR	-	-	20.4		11.6	1.6	-	15.4	5.4	-	-
Beta lactamase sensitive penicillins - J01CE	-	19.1	-	0.8	-	-	-	3.4	5.4	-	3.5
Beta lactamase resistant penicillins - J01CF	-	-	-	3.0	-	1.6	40.3	3.2	1.1	9.3	-
1^st^ generation cephalosporins - J01DB	3.0	24.6	18.3	3.0	8.8	-	-	25.5	5.4	-	26.8
2^nd^ generation cephalosporins - J01DC	-	-	5.8	-	-	-	-	-	1.1	-	
3^rd^ generation cephalosporins - J01DD	-	-	2.6	21.7	19.1	1.6	1.6	19.7	3.2	-	
4^th^ generation cephalosporins - J01DE	-	-	12.2	15.6	-	3.2	-	0.4	1.1	-	
Carbapenem - J01DH	-	-	4.4	8.0	13.1	53.2	-	1.0	2.2	-	5.6
Sulfonamides of action Interm. - J01EC	-	-	-	-	-	-	-	-	1.1	-	2.2
Combination of sulfonamides and trimethoprim - J01EE	-	-	-	0.4	1.9	-	-	0.4	3.2	-	
Macrolides - J01FA	35.0	-	-	-	-	-	-	5.8	7.5	-	-
Lincosamides - J01FF	-	-	2.4	8.0	7.2	-	-	0.4	2.2	-	9.9
Aminoglycosides - J01GB	11.0	20.6	3.6	2.7	6.9	-	-	6.4	3.2	-	6.9
Fluoroquinolones - J01MA	-	-	-	10.6	8.1	-	-	0.6	4.3	-	15.1
Other quinolones - J01MB	-	-	-	-	-	-	1.6	-	1.1	-	-
Glycopeptide - J01XA	-	-	3.5	8.7	8.1	69.4	46.8	3.2	2.2	-	11.2
Polymyxins - J01XB	-	-	-	-	-	46.8	-	0.2	1.1	-	-
Imidazolic derivatives - J01XD	-	-	5.3	-	-	-	-	9.0	-	-	-
Nitrofuran derivatives - J01XE	-	-	-	-	-	-	-	-	2.2	-	-
Other antibacterials - J01XX	-	-	-	-	-	-	1.6	0.6	-	-	-

ICU: intensive care unit; Entire hospital: consumption of the entire hospital; Ped: all sectors that care for pediatrics (infirmary and ICU); Ped ward: pediatric ward; CM: medical clinic; tto emp: empirical treatment; tto dir: targeted treatment; Cl & Sur ward: clinical ward and surgical ward.

Nevertheless, only one study described how the relative frequency of antibiotic prescriptions was calculated (percentage of patients treated with a specific antibiotic divided by the total number of patients treated with antibiotics). In three studies, we determined how the relative frequency of antibiotic prescriptions was measured by calculating the percentage ourselves from the absolute prescription frequency presented in the manuscript (the percentage of a specific antibiotic prescription divided by the total number of antibiotic prescriptions). Furthermore, seven studies described the source of data collection (two adult ICUs, three pediatric wards and ICUs, one pediatric ward, and two adult wards). Only two studies presented the ATC index version Supplementary Material (Table 2).

Regarding the geographic distribution of works published in Brazil, the state of São Paulo was the most represented (five publications); followed by Minas Gerais (four); Paraná, Santa Catarina, Rio Grande do Sul, Distrito Federal, Mato Grosso do Sul (two each); and Pernambuco, Paraíba, Bahia, and Rio de Janeiro (one each). Thus, the South and Southeast regions were the most represented.

Table 5 shows the quality scores of articles included in this systematic review. Only 34.8% of studies received a “yes” answer for the seven questions.

**TABLE 5:** Methodological quality of the articles included (n = 23).

Article	Screening questions		Quantitative non-randomized					Quantitative descriptive				
	S1	S2	3.1	3.2	3.3	3.4	3.5	4.1	4.2	4.3	4.4	4.5
Caldeira et al., 2009^20^	Yes	Yes						Yes	Yes	Yes	Yes	Yes
dos Santos et al., 2007^21^	Yes	Yes						Yes	Yes	Yes	Yes	Yes
dos Santos et al., 2010^22^	Yes	Yes	Yes	Yes	Yes	Yes	Yes					
dos Santos et al., 2013^23^	Yes	No										
dos Santos et al., 2018^24^	Yes	Yes	Yes	Yes	Yes	No	Yes					
EmyInumaru et al., 2019^25^	Yes	Yes						Yes	Yes	No	Yes	Can't tell
Fonseca et al., 2004^26^	Yes	No										
Federico et al., 2018^27^	Yes	Yes						Yes	Yes	Yes	Yes	Yes
Giacomini et al., 2017^28^	No											
Gimenes et al., 2016^29^	Yes	Yes						Yes	Yes	No	Yes	No
Gonçalves et al., 2009^30^	Yes	Yes						Can't tell	Can't tell	Yes	Can't tell	No
Janebro et al., 2008^31^	Yes	No										
Lima et al., 2016^32^	Yes	Yes						Yes	Yes	Yes	Yes	Yes
Marra et al., 2009^33^	Yes	Yes	Yes	Yes	Yes	No	Yes					
Monreal et al., 2009^34^	Yes	No						Yes	Yes	No	Yes	No
Moreira et al., 2013^35^	Yes	Yes	Yes	Yes	Yes	No	Can't tell					
Neves et al., 2010^36^	Yes	Yes						Yes	Yes	Yes	Yes	Yes
Oliveira et al., 2012^37^	Yes	Yes	Yes	No	Yes	No	No					
Rocha et al., 2009^38^	Yes	Yes	Yes	Yes	Yes	No	Yes					
Rodrigues et al., 2010^39^	Yes	Yes						Yes	Yes	Yes	Yes	Yes
Rodrigues et al., 2013^40^	Yes	Yes	Yes	Yes	Yes	No	Yes					
Souza et al., 2008^41^	Yes	Yes	No	Yes	Yes	No	Yes					
Vasconcelos-Pereira et al., 2011^42^	Yes	Yes						Yes	Yes	Yes	Yes	Yes

Among the studies that analyzed antibiotic consumption using the DDD, we found that according to 5 of the 16 results for antibiotic use (each represented by a column in Tables 3 and 4 since a study may have evaluated the consumption of more than one site or treatment), third-generation cephalosporins were not among the three most used classes of antibiotics. Notably, two of them (2/5) were applied to calculate the consumption of antibiotics prescribed for infections caused by sensitive or resistant *Staphylococcus aureus* ^29^ and one for *Pseudomonas aeruginosa* ^36^, while another evaluated only the use of imipenem^41^, and the last one calculated the antibiotic consumption of an entire hospital over four months^39^. 

The high use of fluoroquinolones and combinations of penicillins is noteworthy, as they were the three most commonly used antibiotics according to six and seven results of antibiotic use. Additionally, the class of penicillin combinations was the most widely used in four results of antibiotic use, the most commonly used being amoxicillin/clavulanate and ampicillin/sulbactam (Table 3).

This high use of third-generation cephalosporins and combinations of penicillins was observed in all hospital types and local studies; however, high use of fluoroquinolones was associated with small-, medium-, and large-sized hospitals. In contrast, a high use of carbapenem was observed in extra-large hospitals and ICUs.

No specific class of antibiotics was predominant among the relative frequency of antibiotic prescriptions. The classes of antibiotics that most frequently appeared among the three highest proportions of use per study were broad-spectrum penicillins; penicillin combinations; beta-lactamase-resistant penicillins; first-, third-, and fourth-generation cephalosporins and fluoroquinolones (Table 4).

## DISCUSSION

This review included 23 articles with different methodological designs. The main objective of seven studies was to analyze antibiotic use^20^ ^,^ ^25^ ^,^ ^26^ ^,^ ^28^ ^,^ ^30^ ^,^ ^39^ ^,^ ^42^, while the others presented this data secondary to a main objective, namely: to investigate the relationship between antibiotic consumption and the incidence of resistant bacteria^27^ ^,^ ^35^ ^,^ ^36^, assess indicators of rational drug use in antibiotic prescriptions^34^, assess the adequacy of antibiotic prescriptions by disease or microorganism^31^ ^,^ ^32^, compare antimicrobial therapy with the microorganisms’ sensitivity profile^22^ ^,^ ^26^ ^,^ ^29^ ^,^ ^37^, and assess the impact of implementing interventions in antibiotic consumption^24^ ^,^ ^33^ ^,^ ^40^ ^,^ ^41^.

Despite the different approaches, these articles were included because they evaluated antibiotic use, which allowed us to discuss the Brazilian hospital scenario.

Most study hospitals were part of the Brazilian public health system, called the Unified Health System (UHS), which comprises a public set of actions and services (from the most basic to the most complex) aimed at providing healthcare for the entire Brazilian population^43^.

Third-generation cephalosporins were the most commonly used antibiotic class in the included studies, but were not among the three most used classes in articles that evaluated the treatment of infections caused by a particular bacteria, such as *S. aureus* or *P. aeruginosa* (Table 3). This is justified, since third-generation cephalosporins are not an option for these types of infections (with the exception of ceftazidime in the treatment of *P. aeruginosa*)^29^ ^,^ ^36^ ^,^ ^43^ ^,^ ^44^. Another publication evaluated only the consumption of imipenem^42^.

In a Chilean study covering 15 hospitals with medical clinics and intensive care and surgical units, similar results were observed, since third-generation cephalosporins were the most commonly used^45^. However, this scenario differs from that observed in developed countries. Studies have shown that the antibiotics most commonly used in European and Japanese hospitals are combinations of penicillins with beta-lactamase inhibitors^46^ ^,^ ^47^. In the 2019 report from the European Centers for Disease Prevention and Control, some European countries evaluated antibiotic use from hospital data similar to that of Brazil (high third-generation cephalosporin use), such as Bulgaria, Greece, Hungary, Latvia, Lithuania, Poland, Romania, and Slovakia^48^.

The excessive use of cephalosporins, especially third generation, is associated with the selection of enterobacteria that produce extended-spectrum beta-lactamase (ESBL); this complicates treatment, since the only effective class of beta-lactams is carbapenems^49^. In addition, infections caused by ESBL-producing bacteria are associated with increased length of hospital stay and hospital mortality^50^.

Also noteworthy is the use of antibiotic classes comprised of combinations of penicillins, fluoroquinolone, and carbapenem (Table 3). At least one of these antibiotic classes is among the three most used in all studies except for those that evaluated antibiotic consumption for the treatment of *S. aureus* (penicillins resistant to beta-lactamase, glycopeptides, and other antibacterials^29^) and in the study by Caldeira and Burattini (2009)^20^. The higher use of carbapenem and lower use of fluoroquinolones are associated with the local study (extra-large hospital and ICU).

This high use of carbapenem in extra-large hospitals and ICUs was also observed in a Japanese (extra-large hospital) study and the Chilean studies (ICU)^45^ ^,^ ^47^. This pattern is explained by the advanced medical care provided in extra-large hospitals. Moreover, ICUs treat patients in critical conditions with a high risk of infections by multiresistant bacteria^51^ ^,^ ^52^.

Carbapenem antibiotics have a broad spectrum of action, including gram-positive cocci, fermenting and nonfermenting gram-negative bacilli, and gram-positive and -negative anaerobes. In addition, the carbapenem class is one of the few options for treating infections caused by ESBL-producing bacteria, and as a consequence of these increased infections, its use has become increasingly more frequent. As a result, the occurrence of carbapenem-resistant bacteria, such as carbapenem-resistant *Enterobacteriaceae*, has been more prevalent over the past decade, with few therapeutic options remaining^53^.

Combined with the use of the DDD (Table 3), some studies applied the percentage of antibiotic prescriptions (Table 4). These drug utilization metrics consider different variables when measuring drug consumptions. The consumption of antibiotics calculated by the DDD considers the number of patients who used antibiotics, the dose, and the length of treatment, whereas the analysis by the percentage of antibiotic prescriptions does not consider the dose or the length of treatment. Furthermore, most studies did not explain how this metric was calculated. Therefore, use of these metrics resulted in different patterns of antibiotic use; however, a comparison of studies included in this review with other studies was unfeasible.

In a systematic review undertaken by Zanichelli et al. (2018)^54^, whose objective was to evaluate the antibiotic utilization metrics in studies carried out worldwide and published in English, 75 studies carried out in institutions with inpatients were included, 28% (21) of which used the percentage of antibiotic prescriptions as a drug utilization metric. However, among the studies that used the percentage, 90.5% (19) were point prevalence studies (studies developed through data collection corresponding to 24 hours of hospitalization) and the other two were carried out in long-term care institutions for the elderly.

The DDD is a drug utilization unit developed by the WHO and is defined as the average daily dose of a drug for its main indication in adults. Thus, use of the DDD is important, as it is a globally standardized drug utilization metric that allows comparison of multiple studies. Although the DDD is a globally standardized indicator, it alone does not provide complete information about how the drugs are used in a given location. Although the DDD considers the length of treatment, number of people who use them, and prescribed dose; however, it does not clarify which of these most significantly influences total consumption. Therefore, it is suggested that studies on antibiotic use choose more than one type of drug utilization metric, such as the average daily dose prescribed, days of treatment, and cost^54^ ^-^ ^56^.

Considering the 23 studies included in this review, only two drug utilization metrics were used to assess antibiotic use: the DDD and the percentage of antibiotic prescriptions. Moreover, only one study analyzed antibiotic consumption using two metrics^22^. Notably, the assessment was made only in the ICU; no Brazilian study evaluated the general consumption throughout a hospital using more than one metric.

Even among studies that adopted the DDD as a consumption metric, the comparison of results is questionable, because some antibiotic classes were revised in the DDD over the years. Few studies have clarified which version was used; however, using the year of publication, it is possible to infer which different version may have been applied. This reinforces the importance of using more than one drug utilization metric. Other factors that hinder comparisons include the lack of standardization in the adoption of the DDD/bed-day, patient-day, or admissions; moreover, some manuscripts even describe how the calculation was performed. Additionally, one study used the DDD for pediatric patients^39^; however, the DDD is the average daily dose for adults and therefore not applicable to a pediatric population. This highlights the importance of standardizing drug utilization metrics and methodologies in studies of antibiotic use in Brazil, which would enable more reliable comparisons.

The application of the DDD as an antibiotic utilization metric is common in countries around the world. Europe, the United States, Japan, and Chile frequently report antibiotic consumption based on well-designed studies presenting complete information for the whole country that allows for comparisons and identification of problems that need interventions to improve antibiotic use^45^ ^-^ ^48^.

In the present review, in addition to evaluating the prevalence of articles that adopted insufficient drug utilization metrics to determine the pattern of antibiotic use, we attempted to compare them with more reliable international studies, but their quality was unsatisfactory according to the MMAT criteria. The main reasons for this low quality were the data collected and measurements used, which did not allow us to address the research question or the confounders unaccounted for in the study analysis. The absence of studies of excellent quality may indicate a lack of training or an incentive to register, analyze, and publish data on antibiotic consumption in hospitals, as well as the difficulty Brazilian researchers have in obtaining financial resources to conduct high-quality, well-designed, multicenter studies^57^.

This systematic review obtained data on the use of antimicrobials in municipalities in 11 Brazilian states, covering four national regions (Southeast, South, Midwest and Northeast), with a prevalence of studies carried out in the Southeast and South (69.6%). These differences probably reflect the greater investment in science and research in regions where most of the country’s research centers are concentrated, but they may also be a sign of structural differences in organization, coverage, and provision of health services in Brazil.

Inappropriate antibiotic use by means of unnecessary prescriptions, prolonged use, and antibiotics with a broad spectrum of action for conditions that could be otherwise treated with restricted-spectrum drugs increases the risk of resistant bacteria selection. Microbial resistance to antibiotics is one of the fastest growing public health concerns. It is estimated that by 2050, it will be among the most lethal health problems, in addition to generating an additional billion dollars in healthcare costs^58^.

One of the main limitations of this systematic review is the absence of studies published in gray literature. Nonscientific documents on antibiotic use in hospitals may have been published but were not included in this study. These data could make the scenario of antibiotic use in Brazil more realistic. Another limitation of this review is the lack of standardized reliable metrics and methods for assessing consumption, which would provide a clearer dimension of antibiotic consumption in Brazil and facilitate comparisons with the global scenario. Nevertheless, this study computed the available information and presented a scenario still unknown in Brazil: the national pattern of antibiotic consumption.

Studies on the use of antibiotics have become essential to support health managers with consumption data for a region, country, or even internationally. Consumption data obtained from well-designed and -conducted studies may support campaigns, strategies, and interventions at the local or global level to rationally use antibiotics^55^ ^,^ ^56^. However, this can only occur through well-designed studies using precise metrics that provide information from a hospital to a national scenario and consider the care complexities of different hospitals.

The few studies found in this review show antibiotic use with high consumption of broad-spectrum antibiotics, mainly when compared to international drug utilization studies. However, the low quality of the studies, the absence of good antibiotic utilization metrics, and the lack of studies with results covering national patterns hinder the understanding of the actual antibiotic consumption in Brazil and confirm the need for high-quality drug utilization research about antibiotic consumption on a national level.
