# Supplementary material for: Antibiotic use in Brazilian hospitals in the 21st century: a systematic review
Source: Rev Soc Bras Med Trop. 2021 Jun 9;54:e0861-2020. doi: 10.1590/0037-8682-0861-2020 (PMC8282254; doi:10.1590/0037-8682-0861-2020)
Supplement: Supplementary file 1 [file 1678-9849-rsbmt-54-e0861-2020-suppl1.pdf]

SUPPLEMENTARY MATERIAL TABLE 1: Characteristics of the included studies (n = 23).

Author. Year of publication (city, state of origin of the study)[Ref.]	Type of study (according to the author's statement)	Aim of study	Sample*	Description of the data collection location	Study period	Indication (Prophylaxis/ Treatment)
Caldeira et al., 2009 (Cascavel, PR)[20]	Retrospective	To analyze the utilized antimicrobial agents at the University Hospital of Western Paraná and in some of its admission units, as well as analyze the actual expenses with the consumption of these drugs	ND	General practice, medical clinic, surgical, obstetric clinic, oncologic, ICU (clinical and surgical), first aid and other areas of attendance	Jan 1999 – Dec 2004	Prophylaxis and Treatment
dos Santos et al., 2007 (Brasília, DF)[21]	ND	To evaluate the pattern of usage of antibacterial drugs in an adult ICU in a general hospital in Brasilia, Brazil, and to identify which indicators of hospital infections were related to the greater usage of these agents	283	Adult ICU	Jan 2001 – Jun 2004	Treatment
dos Santos et al., 2010 (Brasília, DF)[22]	Prospective cohort	To know and compare the patterns of antibacterial utilization in intensive care units not specialized in Brasília (Brazil)	1,069	Adult ICU	Oct 2004 – Sep 2005	Treatment
dos Santos et al., 2013 (Porto Lucena, RS)[23]	*Feasibility study*	To assess the feasibility of the antimicrobial stewardship programme during a 4-month period	76	-	May – Aug 2011	Prophylaxis and Treatment
dos Santos et al., 2018 (Rio do Sul, SC)[24]	Quasi-experimental	To describe a 2-year telemedicine intervention and its impact on antimicrobial consumption and multidrug bacterial resistance	6163	-	May 2014 – Apr 2016	Prophylaxis and Treatment
EmyInumaru et al., 2019 (Tubarão, SC)[25]	Cross-sectional	To examine the profile and appropriate use of antibiotics among hospitalized children	318	Pediatrics and Pediatric ICU (2 to 11 years old)	Jan – Dec 2015	Prophylaxis and Treatment
Fonseca et al., 2004 (Marília, SP)[26]	Cohort	To determine the percentage of patients who received antibiotics, the drugs most frequently used, the indications for use, and the most frequent types of infections	144	-	6 months**	Prophylaxis and Treatment
Federico et al., 2018 (São Paulo, SP)[27]	Ecological	To evaluate trends and the immediate and late impact of antimicrobial consumption on CRAs, CRPA, and CRKs over a 10-year period	9,988	Medical and surgical wards, solid-organ and bone-marrow transplant and ICU	Jan 2007 – Dec 2016	Prophylaxis and Treatment
Giacomini et al., 2017 (Botucatu, SP)[28]	Ecological	To study the 60-day use of parenteral antimicrobials in 48 hospitals with up to 50 beds in inner Brazil	7325	Acute care hospitals	Mar 2015 – Dec 2016	Prophylaxis and Treatment
Gimenes et al., 2016 (Maringá, PR)[29]	Retrospective observational	To evaluate antibacterial therapy after susceptibility testing of *S. aureus* infections	94	Adult ICU	72 months**	Treatment
Gonçalves et al., 2009 (Belo Horizonte, MG)[30]	Retrospective, descriptive and cross-sectional	To identify the antimicrobial drugs used in the pediatric units of two hospitals and to examine whether the drug dosage forms used were really suitable for pediatric patients	ND	Pediatrics and Pediatric ICU (28 days to 19 years old)	Jan – Dec 2005	Prophylaxis and Treatment
Janebro et al., 2008 (Campina Grande, PB)[31]	Exploratory	To evaluate the penicillin pharmacological therapy in the pediatric area	105	Pediatric ward (1 day to 18 years old)	Dec 2003 – Mar 2004	Prophylaxis and Treatment
Lima et al., 2016 (Recife, PE)[32]	Cross-sectional	To assess the adequacy of antibiotic prescription in children hospitalized for pneumonia in a reference pediatric hospital in Brazil	452	Pediatrics and Pediatric ICU (1 month to 60 months old)	Oct 2010 – Sep 2013	Treatment
Marra et al., 2009 (São Paulo, SP)[33]	Quasi-experimental before and after type	to limit the duration of antimicrobial therapy to 14 days to reduce both antimicrobial agent consumption and bacterial resistance in an ICU	ND	ICU	1st phase: Jan – Oct 2006, 2nd phase: Nov 2006 – Aug 2007	Treatment
Monreal et al., 2009 (Campo Grande, MS)[34]	Descriptive/cross-sectional	Evaluation of Rational Use of Medicines in Antimicrobials Prescriptions in a University Hospital, Brazil	258	Medical and AIDS wards	Aug 2007 – May 2008	Prophylaxis and Treatment

Author. Year of publication (city, state of origin of the study)Ref.	Type of study (according to the author's statement)	Aim of study	Sample *	Description of the data collection location	Study period	Indication (Prophylaxis/ Treatment)
Moreira et al., 2013 (Uberlândia, MG)[35]	Transversal	To evaluate antimicrobial usage, incidence, etiology, and antimicrobial resistance trends for prominent nosocomial pathogens causing ventilator-associated pneumonia in a clinical-surgical ICU	ND	Adult ICU	1st phase: May 2006 – Apr 2007, 2nd phase: Sep 2008 – Aug 2010	Treatment
Neves et al., 2010 (Botucatu, SP)[36]	Ecological retrospective	To investigate correlations between the aggregate use of antipseudomonal drugs and the incidence of MDR-PA among several units from a teaching hospital	350	-	Jan 2004 – Dec 2005	Prophylaxis and Treatment
Oliveira et al., 2012 (Belo Horizonte, MG)[37]	Retrospective, historical cohort type	To evaluate the repercussions of discontinuation the cost with the antimicrobial treatment of patients with bloodstream infection	62	ICU	Mar 2007 – Mar 2011	Treatment
Rocha et al., 2009 (Rio de Janeiro, RJ)[38]	ND	To describe restricted antimicrobial agents´ use patterns in HMCF, during 2003 and 2004	ND	-	Jan 2003 – Dec 2004	Prophylaxis and Treatment
Rodrigues et al., 2010 (Santa Maria, RS)[39]	Descriptive and cross-sectional	To describe the profile of the antimicrobials utilized in a private hospital in the countryside of Rio Grande do Sul State and the quantity of each antimicrobial consumed	483	-	Mar – Jun 2006	Prophylaxis and Treatment
Rodrigues et al., 2013 (Uberlândia, MG)[40]	Prospective intervention study with historical control	To know the effectiveness of an intervention to implement the SAT in a teaching hospital in Brazil	234	Surgery wards, adults ICU, internal medicine wards	Apr - Jul 2005	Treatment
Souza et al., 2008 (Salvador, BA)[41]	Observational before and after	To analyze the use pattern of imipenem following the restructuring of the antimicrobial audit system at a University Hospital	51	Surgical ward, medical ward, infectious diseases ward and ICU	May – Dec 2006	Prophylaxis and Treatment
Vasconcelos-Pereira et al., 2011 (Campo Grande, MS)[42]	Descriptive and cross-sectional	To identify the use of standardized and restricted intravenous antibacterial drugs at the University Medical Centre of Federal University of Mato Grosso do Sul during period of November 2007 to April 2008		Internal medicine (cardiology, rheumatology, neurology and pulmonary medicine), maternity, ICU, coronary care unit, infectious and parasitic diseases, orthopedics, urology, surgical clinic, oncology and emergency medical care	Nov 2007 – Apr 2008	Treatment

Ref: reference; **ICU:** Intensive Care Unit; **DDD:** defined daily dose; **AIDS:** Acquired Immunodeficiency Syndrome; **ND**: not declared: The authors did not report the information; BA: Bahia, DF: Distrito Federal, MG: Minas Gerais, MS: Mato Grosso do Sul, PE: Pernambuco, PB: Paraíba, PR: Paraná, RJ: Rio de Janeiro, RS: Rio Grande do Sul, SC: Santa Catarina , SP: São Paulo, **CRAs:** carbapenem-resistant Acinetobacter spp., **CRPA:** carbapenem-resistant Pseudomonas aeruginosa, **CRKs:** carbapenem-resistant Klebsiella spp., **MDR-PA:** multidrug-resistant *Pseudomonas aeruginosa*, **HMCF:** Cardoso Fontes Municipal Hospital, SAT: Sequential antibiotic therapy.*number of patients or prescriptions. **study period not clearly described.
