# Supplementary material for: Antibiotic use in Brazilian hospitals in the 21st century: a systematic review
Source: Rev Soc Bras Med Trop. 2021 Jun 9;54:e0861-2020. doi: 10.1590/0037-8682-0861-2020 (PMC8282254; doi:10.1590/0037-8682-0861-2020)
Supplement: Supplementary file 2 [file 1678-9849-rsbmt-54-e0861-2020-suppl2.pdf]

SUPPLEMENTARY MATERIAL TABLE 2: Characteristics of the data from studies included (n = 23).

Author. Year of publication (city, state of origin of the study)[Ref.]	Antibiotics included	Patients	ATC/DDD version	Measurement unit for consumption	DDD calculation	Relative frequency calculation (%)
Caldeira et al., 2009 (Cascavel, PR)[20]	Amikacin, cefotaxime, ceftazidime, ceftriaxone, ciprofloxacin, clindamycin, imipenem/cilastatin, oxacillin and vancomycin	All inpatients who were treated with antibiotics	1999	DDD/100 patients-day	DDD number (sum of grams converted to DDD)/number of patients-day (OxNxT)	NA
dos Santos et al., 2007 (Brasília, DF)[21]	All drug belonging to J01 ATC classification	All inpatients who were treated with antibiotics	2005	DDD/1.000 patients-day	DDD number (sum of grams converted to DDD)/patients-day	NA
dos Santos et al., 2010 (Brasília, DF)[22]	All drug belonging to J01 ATC classification	-	2005	DDD/1.000 patients-day and patient frequency	DDD number (sum of grams converted to DDD)/patients-day	Number of patients treated with specific antibiotic/ number of total of patients treated with antibiotics
dos Santos et al., 2013 (Porto Lucena, RS)[23]	-	-	-	Patient frequency	NA	-
dos Santos et al., 2018 (Rio do Sul, SC)[24]	1st-, 2nd-, 3rd- and 4th- generations cephalosporins, penicillin with beta-lactamase inhibitors; quinolones; aminoglycosides; carbapenems and vancomycin	All inpatients who were treated with antibiotics	-	Patient frequency	NA	-
Emylnumaru et al., 2019 (Tubarão, SC)[25]	-	All inpatients who were treated with antibiotics	-	Patient frequency	NA	-
Fonseca et al., 2004 (Marília, SP)[26]	-	All inpatients who were treated with antibiotics	-	Patient frequency	NA	-
Federico et al., 2018 (São Paulo, SP)[27]	All drug belonging to J01 ATC classification	All inpatients with *Klebsiella* spp., *Pseudomonas aeruginosa* and *Acinetobacter* spp	-	DDD/100 patients-day	-	NA
Giacomini et al., 2017 (Botucatu, SP)[28]	Aminoglycosides, fluoroquinolones, aztreonam, ceftazidime, cefepime, vancomycin, piperaciline-tazobactam, carbapenems, polymyxins, linezolid and daptomycin	All inpatients who were treated with antibiotics	-	DDD/100 admissions	-	NA
Gimenes et al., 2016 (Maringá, PR)[29]	All drug belonging to J01 ATC classification	All inpatients who were admitted in ICU and tested positive for *Staphylococcus aureus*	-	DDD/1.000 patients-day	DDD number (sum of grams converted to DDD)/patients-day	-
Gonçalves et al., 2009 (Belo Horizonte, MG)[30]	All drug belonging to J01 ATC classification	All inpatients who were treated with antibiotics	2006	Patient frequency	NA	-
Janebro et al., 2008 (Campina Grande, PB)[31]	All penicillins	All inpatients who were treated with penicillins	-	Patient frequency	NA	-

Author. Year of publication (city, state of origin of the study)[Ref.]	Antibiotics included	Patients	ATC/DDD version	Measurement unit for consumption	DDD calculation	Relative frequency calculation (%)
Lima et al., 2016 (Recife, PE)[32]	-	All inpatients diagnosed with pneumonia	-	Patient frequency	NA	-
Marra et al., 2009 (São Paulo, SP)[33]	-	All inpatients who were treated with antibiotics	-	DDD/1.000 patients-day	-	NA
Monreal et al., 2009 (Campo Grande, MS)[34]	All drug belonging to J01 ATC classification	All inpatients who were treated with antibiotics	-	Patient frequency	NA	-
Moreira et al., 2013 (Uberlândia, MG)[35]	Vancomycin, teicoplanin, imipenem, meropenem, ertapenem, ceftriaxone and cefepime	All inpatients diagnosed with ventilator-associated pneumonia	-	DDD/1.000 patients-day	-	NA
Neves et al., 2010 (Botucatu, SP)[36]	Amikacin, ciprofloxacin, ceftazidime and imipenem	All inpatients who had at least one clinical culture positive for *Pseudomonas aeruginosa*	-	DDD/1.000 patients-day	-	NA
Oliveira et al., 2012 (Belo Horizonte, MG)[37]	-	All inpatients diagnosed with bloodstream infection caused by *Staphylococcus aureus*	-	Patient frequency	NA	-
Rocha et al., 2009 (Rio de Janeiro, RJ)[38]	Carbapenems, glycopeptides, fluoroquinolones, 2nd-, 3rd- and 4th- generations cephalosporines, combinations of penicillins, macrolides, polymyxins	All inpatients who were treated with antibiotics	2005	DDD/100 beds-day	-	NA
Rodrigues et al., 2010 (Santa Maria, RS)[39]	-	All inpatients who were treated with antibiotics	2006	DDD/100 beds-day	DDD from a specified antibiotic x period of observed time x available beds x beds occupancy rate	NA
Rodrigues et al., 2013 (Uberlândia, MG)[40]	-	-	-	Patient frequency	NA	-
Souza et al., 2008 (Salvador, BA)[41]	Imipenem	All inpatients who were treated with imipenem	-	DDD/100 beds-day	-	NA
Vasconcelos-Pereira et al., 2011 (Campo Grande, MS)[42]	Cefepime, cefotaxime, cefoxitin, ceftazidime, ceftriaxone, ciprofloxacin, imipenem + cilastatin, levofloxacin, linezolid, meropenem, piperacillin + tazobactam, polymyxin B, teicoplanin and vancomycin	-	2010	DDD/100 beds-day	Sum of a specified antibiotic grams used in a period of time/DDD of specified antibiotic x period of time x beds occupancy rate	NA

-: missing information; **ATC:** anatomical, therapeutic chemical classification system; **DDD:** Defined Daily Dose; **O:** specific rates of beds occupation of the service; **N:** number of available beds; **T:** time period in days; **ICU:** intensive care unit; **NA:** not applicable.
